# Effects of fatty and lean fish intake on stroke risk: a meta-analysis of prospective cohort studies

**DOI:** 10.1186/s12944-018-0897-z

**Published:** 2018-11-23

**Authors:** Zhi-Zhen Qin, Jia-Ying Xu, Guo-Chong Chen, Yu-Xia Ma, Li-Qiang Qin

**Affiliations:** 1 School of Public Health, Hebei Medical University, 361 East Zhongshan Road, Shijiazhuang, 050017 Hebei Province China; 2 State Key Laboratory of Radiation Medicine and Protection, School of Radiation Medicine and Protection, Soochow University, 199 Ren’ai Road, Suzhou, 215123 Jiangsu Province China; 3 Department of Nutrition and Food Hygiene, School of Public Health, Soochow University, 199 Ren’ai Road, Suzhou, 215123 Jiangsu Province China

**Keywords:** Fatty fish, Lean fish, Stroke risk, Meta-analysis

## Abstract

**Background:**

Fish intake has been postulated to reduce the risk of stroke. However, whether the beneficial effect of fish are mainly linked to fat content, as a source of omega-3 polyunsaturated fatty acids, remains unclear. We conducted a meta-analysis to compare the effect of fatty and lean fish intake on stroke risk.

**Methods:**

We performed a literature search on four database (PubMed, Embase, Scopus, and Cochrane Library) through February 1, 2018 to identify prospective studies of fatty and lean fish in relation to stroke risk. A random-effects model was used to calculate the summary estimates.

**Results:**

We identified five prospective studies, including 7 comparisons for fatty fish intake and 5 comparisons for lean fish intake. Compared with the highest category of intake with lowest category, the summary relative risk was 0.88 [95% confidence interval (CI), 0.74–1.04] for fatty fish intake and 0.81 (95% CI, 0.67–0.99) for lean fish intake. No heterogeneity across studies and publication bias were observed.

**Conclusion:**

Our findings demonstrated that fatty and lean fish intake has beneficial effects on stroke risk, especially lean fish intake. Additional prospective studies are necessary to confirm these observations.

## Background

Stroke is the major cause of morbidity and mortality. In 2013, stroke was the second most common cause of deaths (11.8% of all deaths) worldwide, and the third most common cause of disability (4.5% of disability-adjusted life-years from all cause) [1]. The risk factors for stroke include family history and genetic backgrounds, improper nutrition, physical inactivity, smoking/tobacco use, and some associated diseases such as hypertension [2].

In 2002, the American Heart Association (AHA) published a scientific statement, “Fish Consumption, Fish Oil, Omega-3 Fatty Acids, and Cardiovascular Disease” [3]. Recently, AHA science advisory further addressed the specific effect of omega-3 polyunsaturated fatty acids (PUFAs) from seafood supplementation on clinical cardiovascular events [4]. Agencies and organizations in different countries advise that at least two servings of fish, especially fatty fish, per week are needed to promote cardiovascular health in the general population [4, 5]. However, evidence was mainly derived from the studies which focus on the beneficial effects of omega-3 PUFAs, not from fatty fish rich in these fatty acids.

Previous meta-analyses showed that fish intake has a protective effect against stroke, especially in ischemic stroke [6, 7]. However, a recently systematic assessment indicated that increasing omega-3 PUFAs has no effect on stroke risk [RR = 1.06, 95% confidence interval (95% CI): 0.96–1.16] [8]. The content of omega-3 PUFAs ranged widely in different kinds of seafood. Fatty fish such as anchovies and herring [eicosapentenoic acid (EPA) + docosahexaenoic acid (DHA): 2300–2400 mg/4 oz], tuna (1700 mg/4 oz), and salmon (1200–2400 mg/4 oz) have higher levels of omega-3 PUFAs. In contrast, lean fish including shrimp (100 mg/4 oz), lobster, scallops, and cod (200 mg/4 oz) have lower levels [9]. In addition, seafood contains other nutrients, which varied greatly in the lean and fatty fish. Hengeveld et al. also demonstrated that the association of fatty fish and lean fish with stroke is likely to be independent from one another [10]. With the accumulation of epidemical studies [11–19] as well as the above converging evidence, we performed a meta-analysis to elucidate whether the effects of fatty and lean fish intake on stroke risk might be different. Findings from this study might provide theoretical guidance for fatty and lean fish intake for general population.

## Methods

Four database (PubMed, Embase, Scopus, and Cochrane Library) were search through February 1, 2018 using the key words fish and stroke without restrictions. Furthermore, we reviewed the reference list of retrieved articles. Prospective studies were included if they reported hazard ratio (HR)/relative risk (RR) with a corresponding 95% CI of stroke relating to fatty or lean fish intake in the healthy population. The data that we collected included the last name of the first author, year of publication, country of origin, study name, number of participants and cases, range or mean of participants’ age, duration of follow-up, covariate adjustments, and maximally adjusted risk estimates and 95% CI of lean and fatty fish intake. We used the Newcastle-Ottawa Quality Assessment Scale (NOS), with the highest scale is nine, to evaluate the quality of each cohort [20]. Study selection and date extraction were conducted independently by two authors (Q-ZZ and X-JY), with any disagreements resolved by consensus.

We estimated the summary risk estimates of stroke for fatty and lean fish intake compared the highest with the lowest category using a random-effects model [21]. Heterogeneity test was performed using Q and *I*^2^ statistics [22]. For the Q statistics, *P* < 0.1 was considered statistically significant; and for the *I*^2^ statistics, the following conventional cutoff points were used: < 25% (low heterogeneity), 25–50% (moderate heterogeneity), and > 75% (severe heterogeneity). We conducted a sensitivity analysis to investigate the influence of a single study on the overall risk estimates by omitting one study in each turn. To assess the potential publication bias, we performed Begg’s and Egger’s tests, and inspected the funnel plots as well [23]. All statistical analyses were performed using STATA software, version 11.0 (STATACorp, College Station, TX, USA). Except where otherwise specified, a *P* value < 0.05 was considered to be statistically significant.

## Results

**Fig. 1:**
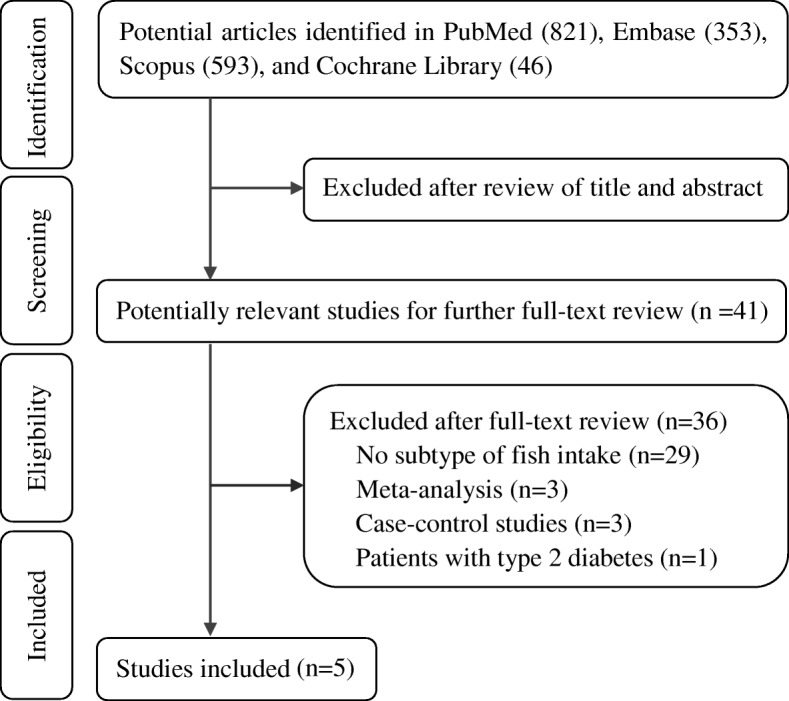
Flow diagram of systematic literature search

The systematic literature search yielded 821 potentially relevant records. After the exclusion of clearly irrelevant publications by reading titles and abstracts, we obtained 41 full articles of potentially relevant studies for further evaluation. After full-text reviews, 29 articles were excluded because of lack of subtype of fish intake. Three studies were excluded because of meta-analysis of total fish intake and stroke risk [6, 7, 24]. Other three studies were excluded because of case-control design [11–13]. One article was further excluded because the participants were patients with type 2 diabetes [14]. Finally, five independent prospective cohort studies with 7 comparisons for fatty fish intake [15–19] and four studies with 5 comparisons for lean fish intake [15–18] were included (Fig. 1).

Characteristics of the included studies are reported in Table 1. Five studies were conducted in the UK (*n* = 2), Italy (*n* = 1), Spain (*n* = 1) and Sweden (*n* = 1). Numbers of participants ranged from 2710 to 41,020. The number of stroke events ranged from 66 to 1680. Except Atkinson’s study [17], other 4 studies excluded participants with a history of cardiovascular disease (CVD) or stroke at baseline. All studies included ischemic and hemorrhagic stroke cases, and the former was the majority of stroke type. The follow-up duration ranged from 4.3 to 18 years. Fish intake was measured by food frequency questionnaire in all studies. Three studies reported fish intake as times/week [15, 18, 19]. One study reported it as grams/day [16], and the last one did not provide the data of fish amounts [17]. Fish intake in the highest category varied largely across each study. Quality scores based on the NOS ranged from 6 to 9.

**Table 1:** Characteristics of the studies included in the meta-analysis

Study	Location	Study name	Length of follow up	Participants	Gender (men %)	No. of cases/size	Quantile	Adjusted RR (95% CI)	
								Fatty fish	Lean fish
Bonaccio, 2017 [15]	Italy	Moli-sani study	4.3	General population aged ≥35 years (mean 54.7 years)	46.0	66/20969	3	0.69 (0.24–1.94)	0.91 (0.30, 2.75)
Amiano, 2015 [16]	Spain	EPIC^a^-Spain	13.8	Participants were mostly blood donors (55–60%) aged 20–69 years	37.8	674/41020	5	0.97 (0.67–1.42) ^b^ 1.30 (0.87–1.94) ^c^	0.84 (0.55–1.29) ^b^ 1.03 (0.65–1.65) ^c^
Atkinson, 2011 [17]	Caerphilly, UK	Caerphilly Prospective Study	18	Representative population samples aged 45–59 years (mean 53.3 years)	100	225/2710	5	0.66 (0.41–1.05)	0.92 (0.57–1.51)
Larsson, 2011 [18]	Sweden	Swedish Mannograpy cohort	10.4	Population-based mammography screening women aged 49-83 years (mean 61.4 years)	0	1680/34670	4	0.94 (0.68–1.29)	0.67 (0.49–0.93)
Myint, 2006 [19]	Norfolk, UK	EPIC-Norfolk	8.5	Free-living population aged 40–79 years (mean 58.7 years)	45.1	421/24312	2	0.88 (0.65–1.19) ^b^ 0.69 (0.51–0.94) ^c^	

^a^European Prospective Investigation into Cancer and Nutrition

^b^Men

^3^Women

**Fig. 2:**
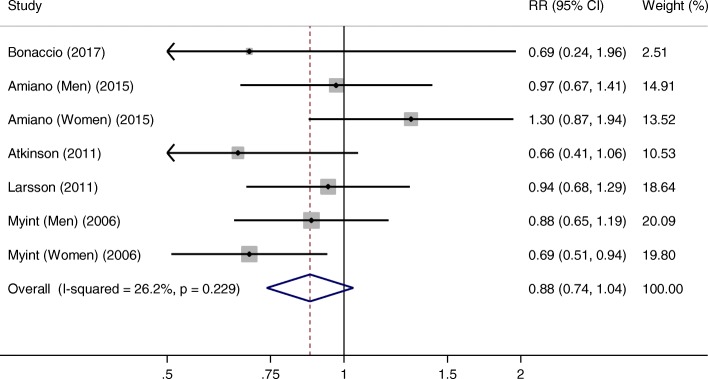
Meta-analysis of the highest versus lowest fatty fish intake and stroke risk

Five studies with 7 comparisons were included in the highest versus lowest analysis of fatty fish intake and stroke. The summary RR was 0.88 (95% CI, 0.74–1.04, *P* = 0.124), with low heterogeneity across studies (*I*^2^ = 26.2%, *P*_heterogeneity_ = 0.229) (Fig. 2). The sensitivity analyses showed a range from 0.83 (95% CI, 0.71–0.96) to 0.93 (95% CI, 0.79–1.10). When Amiano’s study [16] on women was excluded, the inverse association of fatty fish intake with stroke risk became significant.

**Fig. 3:**
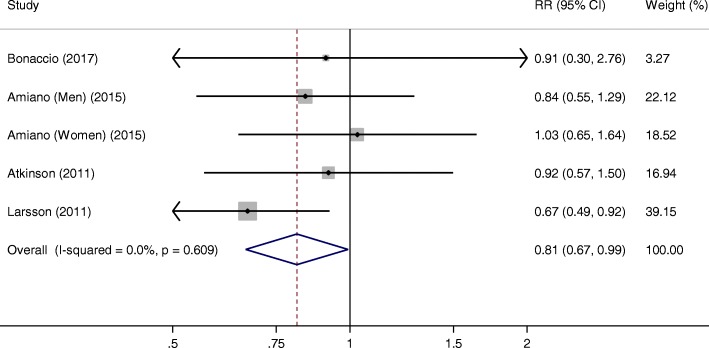
Meta-analysis of the highest versus lowest lean fish intake and stroke risk

Four studies with 5 comparisons were included for lean fish intake and stroke risk. The summary RR was 0.81 (95% CI, 0.67–0.99, *P* = 0.042), without heterogeneity across studies (*I*^2^ = 0%, *P*_heterogeneity_ = 0.609) (Fig. 3). The sensitivity analyses showed a range from 0.77 (95% CI, 0.62–0.96) to 0.92 (95% CI, 0.79–1.19). The summary effect size became non-significant when Larsson’s study [18] was excluded.

There was no obvious dissymmetry in the shape of funnel plots for both analyses of fatty and lean fish intake. Publication bias was also not found using Begg’s test (*P* = 0.764 for fatty fish, *P* = 0.462 for lean fish) and Egger’s tests (*P* = 0.891 for fatty fish, *P* = 0.324 for lean fish).

## Discussion

Our meta-analysis was the first to analyze the effect of fatty and lean fish intake on stroke risk. A significantly beneficial association was observed between lean fish intake and stroke risk. A lower stroke risk by fatty fish intake did not obtain statistical significance.

Our findings were somewhat opposite to the general knowledge that fatty fish is “better” than lean fish. The health benefits of fish intake have traditionally been attributed to the effect of PUFAs, such as EPA and DHA [6, 13, 17, 19]. However, a meta-analysis including 9 randomized controlled trials (RCTs) showed that no statistically significant association was observed with stroke when combined studies supplemented PUFAs [25]. In fact, advantages associated with PUFAs were mainly observed for high amounts, often not within the range of dietary intake of fish [15]. Thus, a recent science advisory from the AHA demonstrated that there is no proven benefit of PUFA supplementation as a means to reduce the risk of stroke [4]. Aside from a variety of nutrients, fish in some cases contains noxious agents. Environmental pollutants, such as persistent organic pollutants and organic mercury, are fat soluble and therefore found mainly in fatty fish. Fish intake was positively correlated with erythrocyte mercury content and EPA + DHA [11]. Both persistent organic pollutants and organic mercury are potential risk factors for CVD and stroke [26, 27]. Daneshmand even found that higher serum omega-3 PUFA concentration was associated with an increased risk of ischaemic stroke among participants with hair mercury content above the median [28].

Lean fish, which contains relatively low amounts of PUFAs, is considered a superior source of proteins. Fish proteins are easy to digest and rich in essential amino acids. Aadland performed an RCT to compare the effect of different protein sources on cardiovascular lipid risk factors in healthy subjects. They found that lean seafood reduced serum triacylglycerol and prevented an elevation in medium-sized, very low-density lipoprotein particle concentrations relative to those of non-seafood intervention [29]. Compared with fatty fish, lean fish contains more iodine, selenium and less energy, which are beneficial to human health [30, 31]. Moreover, lean fish intake significantly decreased circulating soluble intercellular cell adhesion molecule-1 concentrations in patients with ischemic heart disease, but fatty fish intake had no effect [32]. Dietary advice in these recommendations emphasize intake of fatty fish due to its high levels of omega-3 PUFA. However, lean fish contains numerous nutrients that may be beneficial in the prevention of CVD, indicating that also lean fish should be included in the diet when targeting the modifiable risk factors of CVD [31].

Some case-control studies observing the effect of fatty and lean fish on stroke risk also deserve consideration. In a nested case-control study, stroke risk in men increased with fatty and lean fish intake [11]. Using the same cohorts with a larger population and a more extensive questionnaire dataset, they found that previous association between high lean fish intake and stroke risk in men could not be repeated [12]. A relatively high proportion of male with high intake of lean fish were single men, who consumed more semi-finished products. In Sweden, semi-finished lean fish product is commonly fried with relatively large quantities of fat. In another case-control study, ischemic stroke risk decreased with fatty fish intake. However, lean fish intake had no effect on ischemic stroke risk in men and increased risk in women [13].

At the human population level, fish consumption could positively affect plasma lipid profiles, consequently decreasing stoke risk. In a Norwegian cross-sectional study, increased intake of fatty and lean fish was associated with decreased serum triglyceride and increased high-density lipoprotein cholesterol [30]. A RCT also indicated that omega-3 PUFA supplementation reduced plasma triglyceride in pre-menopausal women [33]. Circulating omega-3 PUFA are considered as possible biomarker for intake of fatty acids. Saber measured circulating fatty acids at baseline in 3 separate US cohorts and found that higher circulating levels of DHA were inversely associated with incident atherothrombotic stroke and DPA with cardioembolic stroke [34]. Arachidonic acid (AA), a chief source of omega-6 PUFAs, is a source of mediators that cause inflammation in vessels. High serum ratio of EPA to AA (EPA/AA) levels may be a good biomarker for the risk of CVD [35]. In a population-based prospective cohort study, a lower serum EPA/AA ratio was significantly increased the risk of coronary heart disease, but there was no evidence of an association with stroke [36].

Although there were no animal studies investigating the effect of fish intake on stroke risk, some stroke-related animal models were used to observe the role of omega-3 PUFA. When rats were intraperitoneally pretreated with DHA before being subjected to focal cerebral cerebral ischemia/reperfusion injury, brain infarction was obviously alleviated with the decrease in blood-brain barrier disruption, brain edema, and inflammatory cell infiltration [37]. However, EPA + DHA intervention for 5 weeks aggravated the edema and bleeding, and oxidative damage in the brain of rats with intracerebral hemorrhagic stroke [38].

A major strength of our study is that the included studies used a prospective cohort design, which eliminates the possibility of reverse causation and minimizes selection bias. Several limitations should be acknowledged. First, residual confounding remains a concern. Although a wide range of potential confounders related to stroke were adjusted for in the multivariate models, the possibility of residual confounding could not be ruled out. Second, misclassification bias may have weakened the strength of the association. Given that dietary assessment was based on self-administered questionnaires, misclassification of dietary intake is inevitable. Third, fatty and lean fish are in a heterogeneous group. The number and proportion of the types of fish consumed varied among countries. For example, the most commonly consumed fish were cod (29.9%), tuna (16.4%) and salmon (14.2) in the UK. Meanwhile, hake/burbot (35.0%), tuna (15.2%) and cod (12.3) were typically consumed in Spain [39]. In addition, some methods of fish preparation, which have not been considered in this study, may differently affect the stroke risk. For example, fatty fish is commonly eaten salted, and lean fish is usually eaten cooked, roasted, or fried in Sweden [18]. Fourth, Then, the data of plasma fatty acid pattern in fatty and lean fish were unavailable, therefore whether the differential effects of fatty and lean fish on stroke risk might be associated plasma fatty acid pattern remain unclear. Fifth, the sensitivity analyses showed that the results were less stable when Amiano’s study in women on fatty fish intake [16] or Larsson’s study on lean fish intake [18] was excluded. The exact explanation was unclear, but possibly was related to only women included in these two cohorts. In general, women were inclined to suffer from milder strokes than men [19]. Finally, the limited number of included studies made the subgroup analysis impossible. We were not able to assess the potential effect modification of variables, including geographic region, gender and stroke type, on stroke risk. Also, a dose-response analysis was not performed. We could not determine whether the relationship between fatty and lean fish intake and stroke risk had a threshold or be was U- or J-shaped.

## Conclusion

Our findings demonstrated that fatty and lean fish intake had beneficial effects on stroke risk, especially lean fish intake. Considering the limited number of included studies and limitations mentioned above, additional prospective studies with improved methods of estimating the intake of different kinds of fish is warranted to investigate whether fatty and lean fish intake has different effects on stroke risk among the general populations.

## Abbreviations

AHA: American Heart Association
CI: Confidence interval
CVD: Cardiovascular disease
DHA: Docosahexaenoic acid
EPA: Eicosapentenoic acid
HR: Hazard ratio
NOS: Newcastle-Ottawa Quality Assessment Scale
PUFA: Omega-3 polyunsaturated fatty acid
RCT: Randomized controlled trial
RR: Relative risk
